# The Role of Astrocytes in the Neurorepair Process

**DOI:** 10.3389/fcell.2021.665795

**Published:** 2021-05-25

**Authors:** Raphaela Almeida Chiareli, Gustavo Almeida Carvalho, Bruno Lemes Marques, Lennia Soares Mota, Onésia Cristina Oliveira-Lima, Rodrigo Mello Gomes, Alexander Birbrair, Renato Santiago Gomez, Fabrício Simão, Friederike Klempin, Marcel Leist, Mauro Cunha Xavier Pinto

**Affiliations:** ^1^Department of Pharmacology, Federal University of Goias, Goiânia, Brazil; ^2^Institute of Biological Sciences, Federal University of Minas Gerais, Belo Horizonte, Brazil; ^3^Research Division, Vascular Cell Biology, Joslin Diabetes Center and Harvard Medical School, Boston, MA, United States; ^4^Charité – University Medicine, Berlin, Germany; ^5^Konstanz Research School Chemical Biology, University of Konstanz, Konstanz, Germany

**Keywords:** astrocytes, brain damage, neurorepair, synaptogenesis, neurogenesis

## Abstract

Astrocytes are highly specialized glial cells responsible for trophic and metabolic support of neurons. They are associated to ionic homeostasis, the regulation of cerebral blood flow and metabolism, the modulation of synaptic activity by capturing and recycle of neurotransmitters and maintenance of the blood-brain barrier. During injuries and infections, astrocytes act in cerebral defense through heterogeneous and progressive changes in their gene expression, morphology, proliferative capacity, and function, which is known as reactive astrocytes. Thus, reactive astrocytes release several signaling molecules that modulates and contributes to the defense against injuries and infection in the central nervous system. Therefore, deciphering the complex signaling pathways of reactive astrocytes after brain damage can contribute to the neuroinflammation control and reveal new molecular targets to stimulate neurorepair process. In this review, we present the current knowledge about the role of astrocytes in brain damage and repair, highlighting the cellular and molecular bases involved in synaptogenesis and neurogenesis. In addition, we present new approaches to modulate the astrocytic activity and potentiates the neurorepair process after brain damage.

## Astrocytes: A Cell Type With Multiple Roles

Astrocytes are glial cells responsible for homeostasis, nutrition and protection of the central nervous system (CNS) (Nedergaard et al., 2003). They were first described in 1846 by Rudolph Virchow as a population of homogeneous cells that provide support for neuronal functions (Molofsky and Deneen, 2015; Perez-Nievas and Serrano-Pozo, 2018). For more than a century, the role proposed for astrocytes was merely their ability to support CNS cells. Innovative thinking has emerged in the last few decades, when the classic view of astrocytes as passive supporting cells changed to a crucial active element in the CNS (Halassa and Haydon, 2010; Kettenmann et al., 2013; Rusakov, 2015).

Astrocytes exhibit a star-shaped morphology with extensively branched processes terminating in fine structures, called perisynaptic astrocytic processes (PAPs), that structurally and functionally interact with synapses (Araque et al., 1999). In a mouse, a single astrocyte can cover up to 100,000 synapses, whereas in the human brain that number is more than one million (Halassa et al., 2007). This property of astrocytes has been confirmed for different experimental models and different regions of the CNS and peripheral nervous system (PNS) (Wilhelmsson et al., 2006).

Based on their function, distribution and/or morphology, astrocytes can be divided into two subpopulations: fibrous astrocytes and protoplasmic astrocytes (Wang and Bordey, 2008). Fibrous astrocytes are highly prevalent in white matter, have long non-branched processes with endfeet involved in Ranvier’s nodes. The protoplasmic astrocytes are mainly found in the gray matter and their morphology displays branched processes that involve synapses, and whose endfeet cover the blood vessels. Astrocytic processes interact with the elaborate network of synaptic terminals, dendrites, and dendritic spines (Wang and Bordey, 2008).

Fibrous and protoplasmic astrocytes show differences patterns of protein expression, however, glial fibrillary acidic protein (GFAP) is the main intermediate filament expressed in both subtypes (Marin-Padilla, 1995; Wilhelmsson et al., 2006; Brozzi et al., 2009; Saur et al., 2014). It is important to note that different isoforms of GFAP can be expressed in astrocytes, and the additional intermediate filament proteins Nestin and Vimentin may also contribute to the astrocytic cytoskeleton (Wang and Bordey, 2008; Kamphuis et al., 2012). In different neuroinflammatory pathologies (e.g., infection, ischemia, neurodegenerative diseases, and brain trauma), an increase in GFAP expression can be observed (Hol and Pekny, 2015; Pekny et al., 2016; Escartin et al., 2019; Smit et al., 2021). GFAP is commonly used as an astrocyte marker, although it is important to note that some other cell types inside (e.g., ependymal cells) and outside (e.g., hepatic stellate cells) the CNS cells can also have GFAP (Liu et al., 2006; Wang and Bordey, 2008), and that some quiescent astrocytes do not have detectable levels of GFAP (Kuegler et al., 2012).

Astrocytes have multiple functions in the CNS and are fundamental to the dynamics of tissue functioning. First, the classic function attributed to astrocytes is the mechanical support of nervous tissue, which is done through their endfeet which forms a network and help to anchor astrocytes to blood vessels, neurons, and other cells. This strategic positioning of astrocytes with blood vessels and synapses, allows these cells to regulate blood flow according to synaptic demand. In addition, the endfeet can also enhance the physical barrier properties of astrocytic processes, limiting the signal and communication between neighboring synapses and, at the same time, favoring the specificity of neurotransmission (Abbott et al., 2010). In addition to functioning as a physical barrier, astrocytes can also influence the permeability of blood vessels by releasing factors, such as cytokines and vasoactive agents, that control the balance of water, ions and other molecules in the CNS (Abbott et al., 2010; Michinaga and Koyama, 2019). In this way, astrocytes are also part of the blood-brain barrier (Beenhakker and Huguenard, 2010).

Another important function of astrocytes is the regulation of brain metabolism, which is carried out through the extensions of protoplasmic astrocytes that surround neurons, maintaining a microenvironment suitable for their metabolic functions (Cornell-Bell et al., 1990). Astrocytes are sensitive to neuronal metabolic demand through neurochemical signaling, which involves the release of glutamate, ATP, nitric oxide (NO), hydrogen peroxide (H_2_O_2_), potassium through pre- and post-synaptic neuronal terminals (Petzold and Murthy, 2011; Rose et al., 2017). For example, astrocyte metabolism specializes in glutamate uptake and its metabolism to glutamine. Once produced, glutamine is released from astrocytes to neurons, where it can be deaminated into glutamate and used as a transmitter. The energy metabolism of astrocytes is coupled to that of neurons by the lactate shuttle hypothesis. This way, the energy of glucose from the bloodstream is made available to the brain. The glucose uptake depends on the activation of the Na^+^/K^+^ pump on the surface of the astrocytes. The glycolytically- produced lactate is transferred to neurons, where it provides energy by fueling oxidative phosphorylation (Pellerin, 2008).

One of the most important functions of astrocytes is the neurotransmitter uptake and release. The privileged positioning of astrocytes allows an efficient coverage of the synapses, where these cells clear the excess of transmitters and optimize neurotransmission. For instance, the rapid uptake of gamma-aminobutyric acid (GABA) prevents the inhibitory signal from spreading to other regions (Kinney and Spain, 2002). Likewise, the glutamate transporters excitatory amino acid transporter 1 (EAAT1) and excitatory amino acid transporter 2 (EAAT2), capture the neurotransmitter in glutamatergic synapses. Therefore, the removal of glutamate by astrocytes from the synaptic cleft guarantees the functioning of synaptic transmission, placing astrocytes as protagonists, instead of merely supporters of synapses (Kinney and Spain, 2002). In addition, astrocytes participate in the synaptic modulation by releasing neurotransmitters and modulators, such as glutamate, purines (ATP, adenosine, and guanine), GABA and D-serine (Bezzi and Volterra, 2001).

Several studies by Clarke and Barres (2013) showed that astrocytes have essential functions in the formation of functional synapses during the development of the CNS. The first evidence that astrocytes participated in the formation of synapses comes from research done with mouse retinal ganglion cells (RGCs). These cells, when grown in the absence of glial cells, formed very few synapses but when grown in the presence of astrocytes or in astrocyte-conditioned medium (ACM), they were able to form ten times more excitatory synapses with an increased functionality (Barres et al., 1988; Meyer-Franke et al., 1995). With this evidence it became clear that astrocytes secrete signals that control the development of synapses (Clarke and Barres, 2013).

Studies have shown that during embryonic development, the neurons need to be physically contacted by astrocytes before becoming receptive to synaptogenic signals secreted by this cell type. This suggests that there is a multi-step process that occurs during synaptic development, beginning with astrocyte-neuron contact that modifies the neuron’s maturational state making it able to make synapses. There are several contact-mediated signaling transducers described, such as integrin-protein kinase C (Hama et al., 2004), neurexin (Barker et al., 2008), gamma protocadherins (Garrett and Weiner, 2009), and neuroligins (Stogsdill et al., 2017). However, the main astrocyte-associated synapse-regulating pathways identified involve interaction of astrocyte-secreted signals with neurons. These signals have diverse roles, including the induction of synapse formation, alteration in presynaptic and postsynaptic function, via recruitment of receptors, induction of synapse maturation, and even synapse stabilization linked to memory formation (Huang et al., 2019; Chen et al., 2020).

Astrocytes also play a central role in responding to brain damage. During brain damage, significant biochemical and morphological changes are observed (Wilhelmsson et al., 2006; Zamanian et al., 2012; Pekny and Pekna, 2014). Reactive astrocytes show hypertrophy of their main cellular processes and changes in their protein profile (Wilhelmsson et al., 2006; Bardehle et al., 2013; Choi et al., 2014). Fibrous and protoplasmic astrocytes exhibit structural differences in their processes after mechanical injury: The fibrous astrocytes exhibit condensed retracted processes (Sun et al., 2010); In contrast, protoplasmic astrocytes exhibited an increase in the length and complexity of the branch of their processes after injury (Kajihara et al., 2001; Wilhelmsson et al., 2006). There is evidence demonstrating that astrocytes can activate the maturation and proliferation of adult neural stem cells through growth factor production, which is critical for the tissue regeneration after damage (Cornell-Bell and Finkbeiner, 1991).

The current scientific evidence indicates that astrocytes guide the neurorepair process, neurogenesis and are essential to reestablish local homeostasis after brain damage (Acosta et al., 2017). In this review, we summarize the current knowledge about the role of reactive astrocytes in brain repair by highlighting the molecular and cellular bases involved in neurogenesis and synaptogenesis. In addition, we highlight new approaches that increase the activity of glial astrocytes responsible for the recovery from injuries or diseases in the CNS.

## The Role of Astrocytes in Brain Damage and Disorders

During the 1970s, the term “reactive astrocytes” was first forged after the discovery of the intermediate filament protein GFAP (Eng et al., 1971). Based on robust evidence from experimental animals, a definition of reactive astrocytes has been proposed covering four main characteristics: (1) a spectrum of molecular, cellular and functional changes that occur in astrocytes in response to CNS injuries and diseases, (2) they vary with the severity of the lesion, (3) they are regulated by inter and intracellular signaling molecules, (4) and can be beneficial or harmful to neighboring cells (Sofroniew, 2009). Thus, astrocyte reactivity can be classified as mild to moderate, diffuse, or severe (Episcopo et al., 2013).

Reactive astrocytes is a common response to CNS injuries/diseases, encompassing a spectrum of changes ranging from hypertrophy (increased cell size) to cell proliferation (Sofroniew and Vinters, 2010). Indeed, the evaluation of the reactivity of astrocytes in animal models for brain damage and disorders so far has been done through changes in the amounts of GFAP protein and in the level of expression of the GFAP gene (Lewis et al., 1984).

Reactive astrocytes has been evolutionary developed as defensive reaction (Falsig et al., 2008; Schildknecht et al., 2012) and therefore, a variety of inter and intracellular signals can trigger reactive astrocytosis (Eddleston and Mucke, 1993). Cytokines, modulate the function of astrocytes by inhibiting or promoting astrocytosis. The best researched cytokines are interleukin-1beta (IL-1β), gamma interferon (IFNγ) and transforming growth factor beta 1 (TGF-β1) (John et al., 2003). The role of IL-1β in inducing reactive astrocytes has been confirmed by experiments using primary astrocyte cultures, where an increase in IL-1β induced the expression of multiple proinflammatory genes such as the inducible form of nitric oxide synthase (iNOS) and tumor necrosis factor alpha (TNFα) (Liu et al., 1998; Falsig et al., 2006).

**FIGURE 1:**
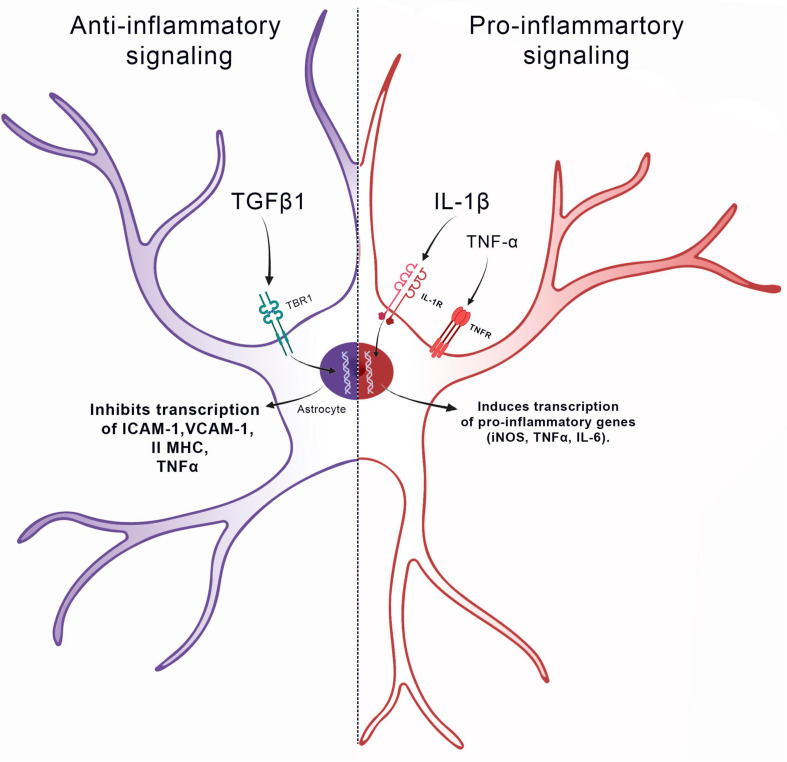
Role of cytokines in promoting astrocytosis. IL1β, IL-6, TNF, and IFNγ induce a pro-inflammatory signaling pathway in the astrocyte, stimulating the gene transcription of iNOS, IL-6, and TNF-α. On other hand, TGF-β1 induces an anti-inflammatory signaling pathway in the astrocyte, inhibiting the gene transcription of ICAM-1, Vcam-1, II MHC, and TNF-α.

TNFα is also markedly up-regulated in injured or inflamed brains in animal models and in humans (Selmaj et al., 1991; Taupin et al., 1993). Furthermore, it has also been shown that the positive regulation of TNFα precedes the increase of GFAP in the brain, a result that correlates this cytokine with the astrocytosis process (Rostworowski et al., 1997). In studies with primary astrocyte culture, it has been shown that the administration of IL-1β can induce the production of TNFα, that activates the transcription of the nuclear factor kappa B (NF-κB), responsible for increasing the production of adhesion molecules, such as intercellular adhesion molecule-1 (ICAM-1) and vascular cell adhesion molecule-1 (VCAM-1) and chemokines, such as interleukin-8 (IL-8) and IP-10 (Lee and Brosnan, 1997; Henn et al., 2011; Kleiderman et al., 2016b; Efremova et al., 2017). Interestingly, TNFα along with IL-1α, IFNγ and C1q are sufficient to induce a markedly pro-inflammatory astrocytic profile, thus confirming the crucial role of these cytokines for promoting astrocytosis (Falsig et al., 2006; Christiansen et al., 2011; Liddelow et al., 2017; Figure 1).

Moreover, although IL-1β, IFN-γ, and TNFα can change the neural microenvironment toward a pro-inflammatory onset (Falsig et al., 2006; Christiansen et al., 2011; Henn et al., 2011; Kuegler et al., 2012; Efremova et al., 2017), transforming growth factor beta 1 (TGFβ1) can play another role in astrocytes. In CNS injury models, inhibition of TGFβ1 activity does not significantly alter GFAP expression but prevents reactive astrocytes from producing glial scarring. This process is responsible for generating a barrier that separates the injured area from healthy nervous tissue (Logan et al., 1994). In addition, the administration of TGFβ1 in primary astrocyte cultures downregulates the expression of several pro-inflammatory genes and induces the expression of multiple extracellular matrices and cytoskeletal molecules. For example, TGFβ1 inhibits the astrocytic expression of major histocompatibility complex class II (MHC class II), ICAM-1 and VCAM-1 molecules, as well as the astrocytic production of TNFα, thus modulating a different pathway for the pro-inflammatory role (Dong and Benveniste, 2001; Figure 1).

Interestingly, the sustained IL-1β signaling also can induce the expression of nerve growth factor (NGF) by astrocytes *in vivo*, which can contribute positively to the maintenance and proliferation of damaged neurons (Sun et al., 2010). In fact, in astrocytic cell culture, IL-1β also induces the expression of a subset of genes including interleukin 6 (IL-6), ciliary neurotrophic factor (CNTF) and NGF, which are primarily associated with neuronal and glial growth and survival. Moreover, activated astrocytes also release other neuroprotective factors like glutathione and cysteine (Gutbier et al., 2018). These data indicate a differential and possibly counterbalance effects of IL-1β signaling in astrocytosis, given that it can promote early inflammatory responses and can trigger regenerative cellular signaling such as the expression of CNTF and NGF. This data indicates that astrocytes, in certain circumstances, become highly cell-protective and beneficial with anti-inflammatory, neuroprotective functions, thus facilitating neuronal recovery and repair (Cornell-Bell and Finkbeiner, 1991; Kajihara et al., 2001; Marchetti and Abbracchio, 2005).

## The Role of Astrocytes in Neurogenesis During Brain Damage

Neurogenesis comprises the set of processes necessary to generate new cells and differentiate them into neurons, which in adult brain, normally is observed in the dentate gyrus (subgranular zone) and in the lateral ventricle (subventricular zone) (Altman and Das, 1965; Eriksson et al., 1998; Alvarez-Buylla and Lim, 2004; Lie et al., 2005; Ming and Song, 2005). Adult neurogenesis is a dynamic process influenced by changes in the microenvironment, such as neurotransmitter release or neurotrophins, but also occurs in response to pathological stimuli (Emsley et al., 2005; Ming and Song, 2005). Neural stem/progenitor cell (NSPC) behavior is affected by seizures and in ischemia and depression; and cells also respond to environmental changes and physical exercises (Kempermann et al., 1997; Kokaia and Lindvall, 2003; Lledo et al., 2006; Parent et al., 2006; Warner-Schmidt and Duman, 2006). Thereby, the specific anatomical and cellular characteristics of the neurogenic niche seems to play an essential role for NSPCs, since they are near to capillary endothelial cells, astrocytes, and ependymal cells (Lim et al., 2000; Palmer et al., 2000; Shen et al., 2004; Ma et al., 2005).

Stem cells of the adult dentate gyrus exhibit radial glia-like properties, express GFAP, and represent the quiescent stem cell population (Alvarez-Buylla et al., 2001). Upon a stimulus, radial glia-like stem cells undergo asymmetric division, whereby they are able to self-renew and generate a progenitor cell to become a neuron or astrocyte (Bonaguidi et al., 2011). The progenitor cells are highly proliferative and amplified and give rise to new neurons (Suh et al., 2007; Goncalves et al., 2016). Surrounding mature astrocytes also help the differentiation and integration of neurons in the hippocampal circuit. Blocking vesicular release of astrocytes has been shown to impair the survival of new neurons and dendritic maturation, reducing branching and the number of dendritic spines in new neurons-that is important for *N*-methyl-D-aspartate (NMDA) receptor functioning-suggesting that astrocytes are important regulators of adult neurogenesis in many stages of the process (Sultan et al., 2015). In addition, β-arrestin-1 (b-arr1) secreted by astrocytes in the dentate gyrus, participates in adult neurogenesis by regulating the production of excretory factors derived from the astrocyte niche and the expansion of precursor cell numbers, thus maintaining homeostasis in the hippocampal niche (Tao et al., 2015).

In the adult brain, astrocytes are not neurogenic under regular conditions, nevertheless, some astrocytes maintain neurogenic potential. The investigation of neurogenic and non-neurogenic astrocytes reveals that distinct subtypes of astrocytes populate different brain areas and present distinct morphological and biochemical features (Bachoo et al., 2004). The majority of astrocytes *in vivo* apparently lose the ability to generate new neurons (Mori et al., 2005). Neural/glial antigen 2 (NG2) positive cells are the major stem/progenitor cell population outside neurogenic regions, that can become neurons *in vitro* (Belachew et al., 2003). In the hippocampus, the brain-derived neurotrophic factor (BDNF) is the main factor involved in the maturation of neurons and in the phenomenon of synaptic plasticity, and thus released by both microglia and astrocytes (Chmielnicki et al., 2004; Ferres-Coy et al., 2013). Nowadays, it is clear that astrocytes can be differentiated in neurons or can release factors that will act in neurogenic niches to stimulate the proliferation and differentiation of NPSCs. Due to that, understanding the difference of glia populations and the factor produced and released during neurorepair may unlock new perspective for adult neurogenesis after brain damage.

The Wnt pathway plays an important role during the development of the CNS and the maintenance of the structure of synapses in addition to the functions of neurons in the mature brain (Clevers et al., 2014; Kumawat and Gosens, 2016; Oliva et al., 2018). Wnt signaling supports the maintenance of cellular homeostasis in the heart and blood vessels, in addition to being the target in some pathologies such as AD, cancer, schizophrenia and multiple sclerosis (van Amerongen and Nusse, 2009; Cerpa et al., 2010; Inestrosa and Arenas, 2010; Gay and Towler, 2017). Currently, there are at least three Wnt signaling pathways: the canonical Wnt/β-catenin pathway, the Wnt/polarity pathway (or planar cell polarity pathway Wnt/PCP pathway) and Wnt/Ca^2+^ pathways (Niehrs, 2012; Humphries and Mlodzik, 2018).

Wnt proteins are generally classified into canonical ligands of the Wnt pathway and non-canonical ligands (Libro et al., 2016). Studies mainly focus on the canonical Wnt/β-catenin signaling pathway in which Wnt signaling depends on the cytoplasmic level of free β-catenin and binds to the transmembrane receptor Frizzled protein (Fz), and to the low-density protein co-receptor related to the lipoprotein receptor (LRP5/6).

Astrocytes in the adult hippocampus express Wnt-3. *In vitro* studies have shown they stimulate Wnt/β-catenin signaling in isolated adult hippocampal progenitor cells (AHPs) and induce the differentiation into neurons. Differentiation induced by a co-culture with astrocytes was reduced in the presence of the soluble Wnt inhibitor of proteins 2 and 3 related to Frizzled- (sFRP2/3) (Lie et al., 2005). In addition to Wnt signaling derived from astrocytes, there is an autocrine Wnt signaling activity in AHPs (Wexler et al., 2009).

**FIGURE 2:**
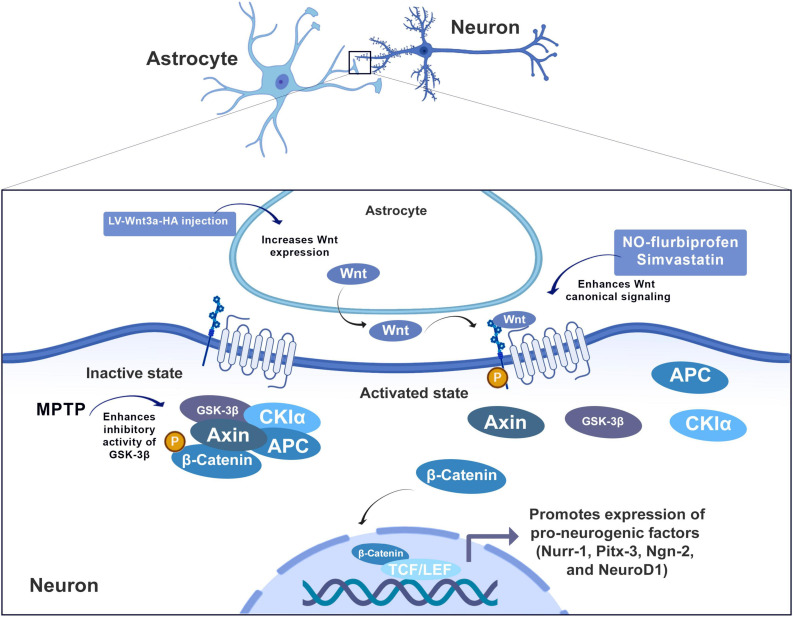
The canonical pathway: Wnt binds to the transmembrane receptor Frizzled protein (Fz) and to the low-density protein co-receptor related to the lipoprotein receptor (LRP5/6) to activate the intracellular Dishevelled protein (Dvl), which therefore inhibits the activity of the complex composed of cytoplasmic glycogen synthase kinase 3β (GSK-3β), adenomatous polyposis coli (APC), and Axin, leading to dephosphorylation, aggregation and nuclear translocation of β-catenin. Within the nucleus, β-catenin combines with T cell transcription factor (TCF)/lymphoid reinforcement factor (LEF) to form a complex, leading to transcriptional activation and expression of specific genes. Wnt/β-catenin signaling enhanced neurogenesis by regulating proneural genes (Nurr-1, Pitx-3, Ngn-2, and NeuroD1).

Wnt/β-catenin signaling enhanced neurogenesis by regulating pro-neuronal genes Nurr-1, Pitx-3, Ngn-2, and NeuroD1 (Singh et al., 2018; Figure 2). Specifically, Wnt-3 and Wnt-3a protein in astrocytes of rats decreased progressively in the dentate gyrus between 2 and 22 months of age, which is accompanied by a decrease in NeuroD1 (Okamoto et al., 2011). NeuroD1, therefore, constitutes a basic helix-loop-helix transcription factor of paramount importance in the generation of granular cells in the embryonic brain (in development) and the mature brain (Gao et al., 2009). We suggest that a decline of Wnt-3/3a in astrocytes may cause a decrease on the expression of pro-neuronal genes and, therefore, a decrease in adult neurogenesis in aging animals (Figure 2).

The Wnt signaling pathway is also involved in Parkinson’s disease (PD). Current evidence points to glial reactivity but whether the activation of glial cells can protect or exacerbate the loss of dopaminergic neurons is currently the subject of debate (Episcopo et al., 2013). The Wnt/β-catenin signaling pathway appears to play a central role in development of dopaminergic neurons in the ventral middle brain (VM) (Gordon and Nusse, 2006; L’Episcopo et al., 2014). The periventricular region of the adult midbrain aqueduct (Aq-PVR) has been shown to harbor neural stem/progenitor cells with dopaminergic potential *in vitro*, but it is believed that restrictive mechanisms *in vivo* limit their regenerative capacity. Using *in vitro* mNPC culture systems, L’Episcopo et al. (2014) demonstrated that aging is a critical factor that restricts neurogenic potential by deregulating Wnt/β-catenin signaling. Co-culture assessments of young-adult progenitor cells and young-adult astrocytes identified a decline in glial-derived factors, including Wnts, while Wnt activation strategies effectively reversed the deficit and enhanced dopaminergic differentiation (L’Episcopo et al., 2014). Astrocytes also participate in the endogenous modulation of regenerative processes through the release of Wnt, which may represent an alternative treatment for CNS injuries, such as ischemia. NSPCs that reside in regions such as the subventricular zone (SVZ) in the adult brain can proliferate and differentiate into other cell types and compensate for the damage caused by ischemia. During development, these cells are largely influenced by the Wnt pathway, according to a previous work by Kriska et al. (2016), where the Wnt pathway/β-catenin is a factor that promotes neurogenesis under the expansion of gliogenesis in neonate mice (Kriska et al., 2016).

In the current studies of this same group, the impact of the Wnt pathway on NSPC modulation was evaluated in transgenic animals and middle cerebral artery occlusion (MCAO) ischemia model animals. In this work, the effects of the Wnt pathway on the modulation of NSPCs were not identified *in vitro* experiments derived from a healthy brain. However, when culturing cells derived from ischemic brains, inhibition of the Wnt pathway resulted in fewer neuron-type cells. In addition, electrophysiology analyzes indicated that blocking this pathway affects the distribution of K^+^ and Na^+^ channels (Kriska et al., 2021). Interestingly, immunohistochemistry analyzes demonstrated an increase in the count of positive cells for labeling Doublecortin (DCX), GFAP and proliferating cell nuclear antigen (PCNA) (Kriska et al., 2021).

Other studies also seek to understand the role of the Wnt pathway in NSPCs after ischemic damage. Wang et al. (2017) demonstrated that some MicroRNAs can play a crucial role in neurological repair, such as miRNA-148b which is overexpressed in the SVZ of ischemic mice. By inhibiting or mimicking miRNA-148b in culture, the effect is to suppress or increase the Wnt/β-catenin pathway. The inhibitor of miRNA-148b promoted the proliferation of NSPCs in neurons and young astrocytes and this action can be silenced with the knockdown of Wnt-1 (Wang et al., 2017). In MCAO models in rats, the injection of this miRNA was able to reduce ischemic damage and improve neurological function (Wang et al., 2017). Another example, Wnt1 levels increased significantly over a period of 1–6 h in the penumbra region in ischemic rats in an MCAO model in the studies by Chong et al. (2010). This fact was also recently described by Jean LeBlanc et al. (2019) demonstrating that β-catenin levels increase in endothelial cells already within 3 h after MCAO model.

Similarly, Wnt3 derived from astrocytes has been related to the differentiation of NSPCs by increasing the expression of synapsin-I and tubulin-III in a paracrine manner. Overexpression of Wnt3 in elderly primary astrocytes using lentivirus expression significantly improved neurogenesis (Okamoto et al., 2011). Interestingly in the SVZ region also the expression of β-catenin and Wnt3a reduced in the subacute phase after ischemia (Wei et al., 2018). Therefore, these studies identify Wnt as a signaling pathway through which astrocytes from specific regions of the CNS regulate adult neurogenesis. In addition, these findings show that Wnts are key regulators of adult neurogenesis *in vivo*, suggesting that they are a central pathway for determining the fate of NSPCs.

The Notch signaling is a common evolutionarily conserved pathway that plays major roles in the regulation of proliferation and differentiation of CNS cells (Lai, 2004; Siebel and Lendahl, 2017). Notch signaling are guaranteed by four different Notch receptors (Notch1-4), which is characterized as a single-pass transmembrane receptor composed by a large extracellular portion and a small intracellular region (Brou et al., 2000). Canonical Notch pathway is mediated by endogenous ligands such as Jagged1-2 and Delta1-4, cell surface proteins that activate the Notch receptor and promotes the proteolytic cleavage -realized by γ-secretase- of the notch intracellular domain (NICD) (Oswald et al., 2001). NICD translocate from the cytoplasm to cell nucleus, where it regulates the expression of several different target genes, mostly Hairy enhancer of split isoforms 1 and 5 (Hes1 and Hes5), through interaction with the RBP-J transcription factor (Jarriault et al., 1995).

Interestingly, there is evidence showing an important role for Notch signaling pathway in promoting reactive astrocyte formation during brain injury. In fact, Shimada et al. (2011) have demonstrated that reactive astrocytes in the peri-infarct area expresses NICD 1, and inhibition of Notch signaling by administration of a γ-secretase inhibitor markedly decrease the number of proliferative reactive astrocytes in this area. Moreover, many different studies have demonstrated an association between Stat3, an important transcription factor that is activated in reactive astrocytes, and Notch signaling. Indeed, it appears that changes in Notch expression can alter Stat3 activity, thus pointing to a modulatory effect of Notch signaling in the promotion of reactive astrocytes (LeComte et al., 2015; Yang et al., 2019).

**FIGURE 3:**
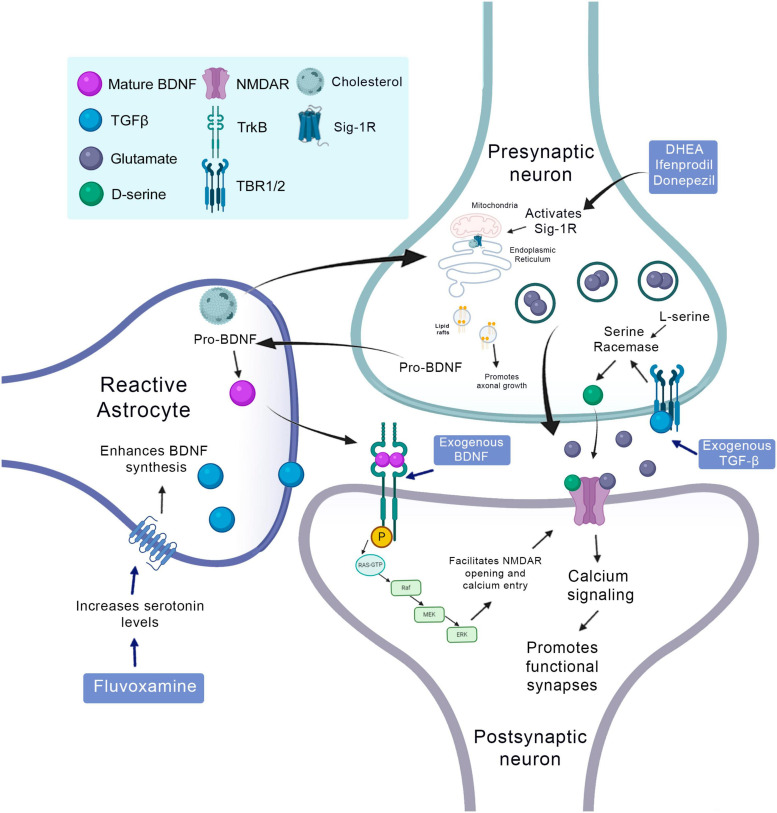
Synaptogenic factors involved in neurorepair in the context of the CNS injury and therapeutic strategies to promote it. Cholesterol released by the astrocyte binds to the sigma-1 receptor and promotes axonal growth. BDNF is produced in the presynaptic neuron and is transformed into mature BDNF in the astrocyte, binds to the TRKB receptor in the postsynaptic neuron and induces its phosphorylation and facilitates the opening of NMDA receptors. The TGF released by astrocytes binds to its TBR1/2 receptor at the presynaptic terminal and activates the serine racemase that converts L-serine to D-serine, which is released in the synaptic cleft and acts on NMDA receptors promoting the formation of functional synapses. The use of fluvoxamine increases the levels of serotonin that will stimulate synthesis and BDNF by the astrocyte. DHEA, ifenprodil and donepezil, are agonists of the sigma-1 receptor. The administration of exogenous BDNF can stimulate phosphorylation of the TRKB receptor and its consequent activity, just as the exogenous administration of TGFβ can stimulate the TBR1/2 receptor.

Another factor secreted by astrocytes during the neurorepair process is D-serine. This amino acid is an important co-agonist of the NMDA receptor. Calcium-dependent release of D-serine by astrocytes participates in long-term potentiation (LTP) of the hippocampus (Henneberger et al., 2010; Sultan et al., 2013; Figure 3). D-serine administration *in vitro* increases the proliferation of stem/progenitor cells of the SVZ and stimulates the survival of new neurons suggesting autocrine regulation (Huang et al., 2012). However, it is still not entirely clear whether the release of D-serine plays a role in regulating adult neurogenesis *in vivo*.

Radzishevsky et al. (2013) report that D-serine is secreted by both astrocytes and neurons, whereby the release of D-serine from astrocytes is triggered by activation of the glutamate receptor agonist α-amino-3-hydroxy-5-methyl-4-isoxazolepropionic acid (AMPA) (Schell et al., 1995). In the hippocampus, the astrocytic niche spans from the hilus over the subgranular zone to the molecular layer, and is therefore ideally located to relay signaling between synaptic activity and the neurogenic niche (Wang et al., 2018).

We have shown above that D-serine, plays an important role in cell proliferation and stimulation of the survival of new neurons. Therefore, it is also important to understand the role of D-serine in a pathological context and how its function implies on neurogenesis and astrocyte function. Psychiatric disorders are characterized by having a polygenic character, that is, they are influenced by distinct genetic variants (Sullivan and Geschwind, 2019). Disrupted-in-Schizophrenia-1 (DISC1) gene is known as risk factor for mental illnesses and present in many psychiatric disorders (Ma et al., 2013). More precisely, DISC1 is considered an important risk factor on neurodevelopment and the use of its mutant form (C-terminus-truncated form) has been used as a molecular tool for the study of the DISC1 pathway in astrocytes (Niwa et al., 2016). Some studies such as Terrillion et al. (2017) reported that an important pathway of DISC1 astrocytes has an important role in stabilizing and binding to the serine racemase (SR) enzyme, which is responsible for the conversion of L-serine to D-serine in astrocytes. The mutant expression of human DISC1 in astrocytes has been shown to reduce endogenous levels of DISC1 in rodents in a negative dominant manner, which results in impaired binding between DISC1 and the serine racemase resulting in increased ubiquitination of the enzyme and reduced production of D-serine by astrocytes. Biochemical changes like these are related several times to an increased response to NMDAR’s non-competitive antagonism by MK-801 which is reversed when in the presence of exogenous D-serine, thus it is suggested that D-serine has a relationship functional with the expression of the DISC1 mutant in astrocytes where the reduction of D-serine production causes changes in behavior (Terrillion et al., 2017).

Other studies have linked DISC1 to maintaining an adequate dendritic morphogenesis of newly formed neurons during adult hippocampal neurogenesis and in the differentiation of newly generated granular cells (Enomoto et al., 2009). In addition, it is shown that astrocytes play a critical role in regulating neurogenesis through D-serine secretion (Sultan et al., 2015). Thus, the hypothesis that the expression of mutant DISC1 in astrocytes would decrease the production of D-serine in the hippocampus is reinforced, leading to a hippocampal neurogenic deficit and consequently behavioral impairments. Specifically, Terrillion et al. (2017) reveal that the expression of mutant DISC1 in astrocytes increased the animals’ anxious behavior, attenuated the social interaction and the preference for social novelty and caused cognitive deficits in rodents. Behavioral changes associated with reduced proliferation of neural progenitor cells and reduced dendritic afforestation of newly formed neurons in the region of the dentate gyrus are observed, which reiterates the data observed when there is a reduction in D-serine in rats with mutation to DISC1. In addition to these observations, treatment with D-serine alleviated the damage in behavior by restoring the typical development of newly formed neurons. Findings like these demonstrate at first glance that the expression of DISC1 in mature astrocytes may be involved in the regulation of neurogenesis and in behavioral parameters that are dependent on the hippocampus activity (Terrillion et al., 2017).

## The Role of Astrocytes in Synaptogenesis During Brain Damage

Synaptogenesis is a process responsible for the formation of synaptic contacts and helps to maintain and eliminate synapses over time. This is a succession of structural events that occurs in neurons that depends on the differentiation of synaptic terminals in specialized membranes that have specific functions. The synapse represents an important functional unit of brain circuits that forms the basis of neural networks. The differentiation of synapses is continually undergoing dynamic changes in response to the needs of the circuits, a process called synaptic plasticity. The effective transfer of information between neurons not only contributes to the formation of functional synapses but can also eliminate those that are unproductive or uncompetitive (Walsh and Lichtman, 2003).

The impairment of neural network function after brain damage is the main cause of disability after brain damage (Desgeorges et al., 2015). Thus, the neurorepair process must be efficient to build a new neural network and reestablish the brain function (Huang et al., 2012). In this context, astrocytes have a critical role in the construction of new synapses and in their maintenance. Astrocytes regulate synapses by direct contact with neurons or secreting soluble factors that act in pre and postsynaptic sites, therefore modulating the structure and function of inhibitory and excitatory synapses (Ullian et al., 2001; Christopherson et al., 2005; Nishida and Okabe, 2007; Stogsdill et al., 2017). Astrocyte structures that make connections with several synapses, are known as PAP. A combination of a PAP with the pre and postsynaptic compartments is known as the tripartite synapse (Araque et al., 1999). Through their fine processes, astrocytes can sense and adhere to synapses and coordinate with the neighboring astrocytes to tile and completely cover the neuropil. Astrocytes do not cover all synapses; however, synapse association of astrocytes is a dynamic process that can be altered by neuronal activity (Genoud et al., 2006; Bernardinelli et al., 2014).

In a tripartite synapse, neurotransmitters released from neurons also bind to receptors on the adjacent PAP, activating signaling pathways in astrocytes that modulate synaptic behavior (Papouin et al., 1715; Araque et al., 1999). In addition to contacting neurons, astrocytes are interconnected by communicating junctions, specialized channels that allow nutrients and ions to diffuse between astrocyte networks, further expanding the range and magnitude of synaptic regulation of neurons by these cells (Pannasch and Rouach, 2013). Recently, the notion of a “tetrapartite synapse,” that includes the extracellular matrix as an important component is emerging (Dityatev and Rusakov, 2011; Smith et al., 2015; Cope and Gould, 2019). Thus, reactive astrocytes can also secrete signals that will assist in the restoration of synapses after CNS injury and can provide a target to promote plasticity and neurorepair (Emirandetti et al., 2006; Tyzack et al., 2014).

Cholesterol-bound apolipoprotein E (APOE) was the first synaptogenic molecule identified by astrocytes. In the CNS, cholesterol is largely produced by astrocytes. It increases presynaptic differentiation in RGC cultures by promoting the synthesis and maturation of synaptic vesicles (Mauch et al., 2001; Goritz et al., 2005). The presynaptic terminals need an adequate number of proteins and lipids to carry out their activities, the binding of cholesterol and synaptophysin, for example, is necessary for the biogenesis of synaptic microvesicles. Cholesterol depletion inhibits this biogenesis probably because it interferes with the formation of the synaptic vesicle curvature (Thiele et al., 2000).

Cholesterol-dependent lipid raft mobilization can help neurorepair through axonal growth after stroke. Stroke-induced increased the cholesterol-binding sigma-1 receptor in astrocytes is beneficial; treatment with a sigma-1 receptor agonist 2 days after MCAO enhances behavioral recovery and neurite outgrowth without decreasing infarct size, suggesting a neural repair, rather than neuroprotection, mechanism (Ruscher et al., 2011). This may be due to increased export of cholesterol to neurons via the sigma-1 receptor. Similarly, increasing high-density lipoprotein cholesterol (HDL-C) by administration of the liver X receptor agonist GW3965 24 h after tMCAO in mice improves recovery, causing synaptogenesis and angiogenesis (Cui et al., 2013). This may partially be explained by the importance of cholesterol in synaptogenesis and neurorepair after injury in the CNS (Gleichman and Carmichael, 2014; Figure 3).

Another molecule secreted by reactive astrocytes during neurorepair processes is thrombospondin (TSPs), which is the main synaptogenic factor secreted by astrocytes (Christopherson et al., 2005). They are large proteins located in the extracellular matrix (Adams and Lawler, 2011). Gray-matter protoplasmic astrocytes express TSP1 and 2, while astrocytes originating in the SVZ and fibrous astrocytes express TSP4 (Eroglu et al., 2009; Benner et al., 2013). The addition of purified TSP in neuron culture increased the number of synapses compared to the number of those with ACM, whereas removal of ACM eliminated most of the synaptogenic activity of ACM. According to these *in vitro* findings, studies using TSP1/2 double knockout (KO) mice showed that these animals exhibited less excitatory cortical synapses, also indicating that TSPs are important for the development of synapses *in vivo*. Interestingly, in the rodent cortex, TSPs are expressed by immature astrocytes only during the first week of postnatal development, a period that corresponds to the beginning of the formation of excitatory synapses on this region (Christopherson et al., 2005).

The receptor to which TSPs bind to induce synaptogenesis is a type 1 transmembrane protein located in neurons, which is a subunit of the calcium channel α2δ-1. This protein has a special domain known as Von Willebrand Factor A (VWF-A) domain, which is the exact place of interaction with TSPs (Eroglu et al., 2009). Therefore, when TSPs bind to this domain, it is believed that this interaction leads to a change in the original conformation of the α2δ-1 receptor, which triggers important processes for the occurrence of synaptogenesis (Risher and Eroglu, 2012). Recently, Risher and colleagues demonstrated in mice the mechanism of action of binding TSPs to the α2δ-1 receptor. It was observed that in the cerebral cortex of these animals this interaction controls synaptogenesis via activation of Rac1 in the post-synaptic neuron, which in turn promotes the remodeling of the actin cytoskeleton in nascent synaptic contact (Risher et al., 2018).

In addition, it has been shown that astrocytes release together with TSPs, another molecule, the innate immune molecule pentraxin 3 (PTX3) (Falsig et al., 2004). This molecule increases the quantity and synaptic grouping of AMPA receptors, therefore playing a key role in promoting functionally active CNS synapses (Fossati et al., 2019). TSPs are the most well-known matrix proteins that are associated with brain tissue repair and synaptogenesis after brain injury. In stroke, TSP-1 is upregulated in the peri-infarct zone within 3 days, while an increase in TSP-2 is observed 1 week later. The relevance of TSP on this process was confirmed by the findings of Liauw et al., 2008 who showed that TSP1 and 2 are necessary for synaptic plasticity and functional recovery of animals after stroke. They induced focal cerebral ischemia in mice and observed that TSP levels increased after brain injury. KO mice for TSP-1 and TSP-2 exhibited deficits on synaptic density and axonal germination when compared to wild animals. They demonstrated that deficits in TSP-1/2 lead to difficulty in recovery after cerebral ischemia mainly due to the role of these proteins on the formation of synapses and in axonal appearance. In addition, they also confirm that TSPs are partially secreted by astrocytes after the stroke, which was evidenced by the co-localization of TSP-1 and TSP-2 with S100 and GFAP, respectively (Liauw et al., 2008; Zhang et al., 2020).

Another article published by Tyzack et al. (2014) further emphasized the importance of TSPs in neurorepair, highlighting a new mechanism for their release. They showed direct evidence that reactive perineuronal astrocytes have great relevance for maintaining the neuronal circuit after distant axotomy. They also revealed a new function as an astrocytic signal transducer and transcription-3 activator (STAT3). STAT3 regulates the formation of the perineuronal astrocytic process and the re-expression of a synaptogenic molecule, TSP-1, in addition to supporting neuronal integrity. It became clear that, through this new route, TSP-1 is responsible for astrocyte-mediated remote recovery from excitatory synapses in axotomized motor neurons in adult mice (Tyzack et al., 2014).

Astrocytes also secrete other proteins important for the formation of new excitatory synapses, such as cysteine-rich acid secreted protein (SPARC) and Hevin, which is also known as SPARC 1-like protein (SPARCL1). These proteins are expressed by the astrocytes of the superior colliculus. Hevin induces the formation of synapses between the RGCs of cultured rats. It is important to note that SPARC antagonizes Hevin’s synaptogenic function (Figure 3; Kucukdereli et al., 2011).

**FIGURE 4:**
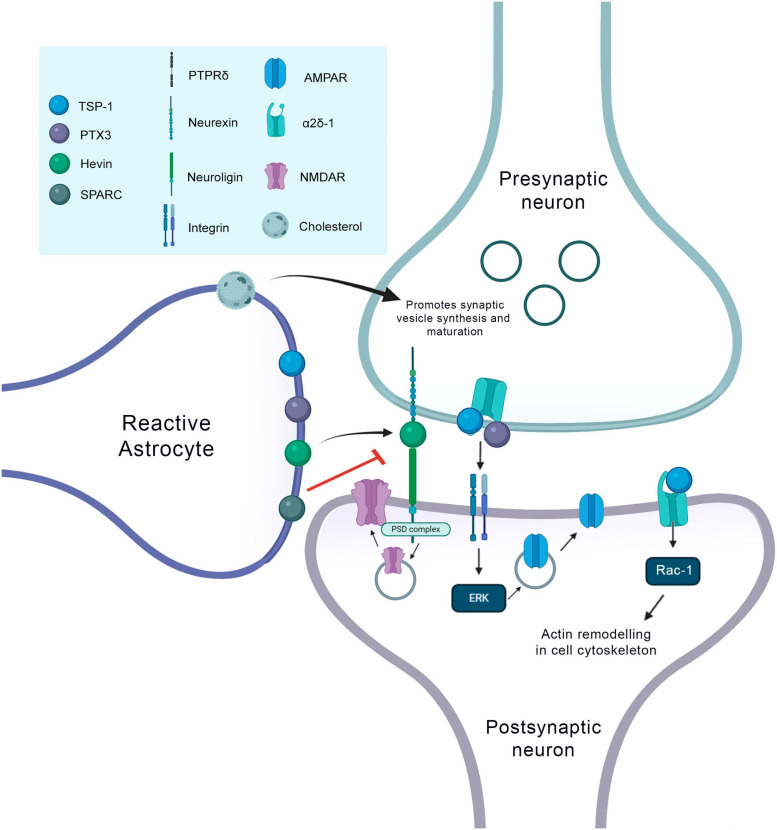
Synaptogenic factors involved in neurorepair in the context of the CNS injury. At the presynaptic terminal, TSP1 and PTX3 bind to the α2δ1 receptor. This binding of TSP at the presynaptic site increases the number of synapses, but they are silent, so the PTX3 molecule released together with TSP, promotes the functional maturation of these synapses by recruiting AMPA receptors. The binding of TSP to postsynaptic receptors, activates the Rac1 protein and stimulates actin remodeling to promote synaptogenesis. Hevin makes the connection between neurexin and neuroligin and thus increases the recruitment of the PSD95 and NMDAR subunits. Hevin’s effect is antagonized by SPARC. Cholesterol increases presynaptic differentiation promoting the synthesis and maturation of synaptic vesicles.

Kucukdereli et al. (2011) have demonstrated that Hevin null mice have fewer excitatory synapses whereas SPARC null mice showed an increase in synaptic connections in the superior colliculus. They concluded that Hevin is a positive regulator and SPARC is a negative regulator of synapse formation and that through the regulation of relative levels of Hevin and SPARC astrocytes can control the formation, maturation, and plasticity of synapses *in vivo* (Kucukdereli et al., 2011). Hevin makes the connection between two neuronal receptors, neurexin at the presynaptic site and neuroligin at the postsynaptic. Neurexin and neuroligin are well known neuronal synaptogenic molecules that can interact with each other. Thus, the presence of Hevin between these two proteins increases the potential for synaptogenesis. In addition, mice without Hevin are unable to remodel synapses in the visual cortex in response to visual deprivation, confirming Hevin’s importance for this type of plasticity during the critical period (Singh et al., 2016). However, a recent study, using rigorous genetic manipulations, showed that Hevin does not require neurexins and neuroligins for his activity, confronting the results mentioned above. Thus, Hevin selectively increases excitatory synaptogenesis and synaptic transmission by a new mechanism that can be independent of neurexins and neuroligins (Gan and Sudhof, 2020; Figure 4).

It is known that after the end of the development of the CNS, there is a reduction in the levels of both Hevin and SPARC. On the other hand, when we have an injury or development of some disease in the CNS, both in reactive astrocytes and in microglia, the amount of these proteins is significantly increased. This was demonstrated in a study developed by Liu et al. (2005), where changes on the production of mRNA and SPARC protein in the hippocampus of adult mice were evaluated after transections of the entorhinal afferents to assess whether SPARC was involved in regulating changes on plasticity induced by adult brain injury. Therefore, they concluded that SPARC was actually involved in denervation-induced neuronal plasticity (Liu et al., 2005). In other studies, a significant increase in Hevin (Lively and Brown, 2007) and SPARC was also observed in reactive astrocytes after injury and stroke (Liu et al., 2005; Lloyd-Burton et al., 2013; Jones et al., 2018).

Neurons in the hippocampus grew more synapses when co-cultured with astrocytes, which is possibly mediated by Agrin – a well-known astrocyte-derived synaptogenesis promoter. It was first described at the neuromuscular junction, as an extracellular matrix protein (Smith and Hilgenberg, 2002; Tournell et al., 2006). Hilgenberg et al. (2006) identified proteins that served as ligands for Agrin, such as membrane tyrosine kinases and the Na/K ATPase.

Some articles discuss the role of protein in synaptogenesis after brain injury, such as a study by Falo et al. (2008), which examined both time and profiles of the Agrin in the deferred hippocampus during reactive traumatic brain injury (TBI)-induced synaptogenesis. They concluded that Agrin increased in lesion-targeted sub-regions, a response associated with synaptic terminals and in sites with reactive astrocytes and that Agrin mRNA transcription was increased in TBI models during the period of rapid synapse formation. In general, these observations identify distinct spatial and temporal differences in Agrin that are associated with the effectiveness of synaptic plasticity. The current results suggest that Agrin plays an important role in the successful synaptic reorganization and neurorepair after TBI (Falo et al., 2008). Recently, the effect of Agrin on neurorepair and synaptogenesis after stroke was investigated. The effect of exercise on this process was also studied. The poststroke exercise improved the recovery of behavioral function and agrin played a critical role in the synaptogenesis process. Due to that, Agrin can be considered a potential therapeutic target for the treatment of stroke and other diseases of the nervous system (Zhang et al., 2020).

Another indication for the synaptogenesis process modulated by astrocytes is the Specificity protein 1 (Sp1). This protein that binds to DNA, can activate or inhibit the transcription of genes in mammalian cells. It belongs to the Krüppel-like protein/factor transcription family (SP/KLF) (Li and Davie, 2010). Several studies show the beneficial role of this protein in relation to diseases or injuries in the CNS, such as in Parkinson’s disease, AD, spinal cord injury and brain trauma. It was also demonstrated that Sp1 has its levels regulated positively in stroke with consequent neuronal protection (Simard et al., 2006; Citron et al., 2015; Miras-Portugal et al., 2016; Wang and Song, 2016; Chuang et al., 2017).

Despite all these studies, the functional role of Sp1 in astrocytes has remained unclear. Recently, Hung and collaborators used Sp1^–/–^ mice to study the function of this protein in astrocytes. They showed that Sp1 can be of great importance for astrocyte function. They found that Sp1 in astrocytes regulates the expression of several genes that are involved in the neuronal development process such as neurite outgrowth and synaptogenesis (Augusto-Oliveira et al., 2020; Hung et al., 2020).

Finally, neurotrophins are molecules of great importance for the development of synapses in both the central and PNSs (Reichardt, 2006; Cohen and Greenberg, 2008). It has been shown in more recent studies that neurons are not the only source of BDNF in the CNS, this neurotrophin can also be found in oligodendrocytes and astrocytes (Dai et al., 2003; Jean et al., 2008). Thus, astrocytes are key elements in BDNF signaling in the brain: pro-BDNF is produced and released by neurons, accumulates in astrocytes, is converted to a mature form of BDNF and is secreted by protein-mediated exocytosis of the membrane associated with the vesicle (VAMP2) (Bergami et al., 2008). BDNF secreted by astrocytes induces phosphorylation of neuronal TrkB, which is essential for the maintenance of LTP and memory retention (Carroll et al., 1993; Emsley and Hagg, 2003). BDNF also regulates the probability of opening the channels at NMDA and GABA receptors, by phosphorylation. NMDA receptors via ErK1/2 and GABA receptors via protein kinase C (PKC) (Jovanovic et al., 2004).

**FIGURE 5:**
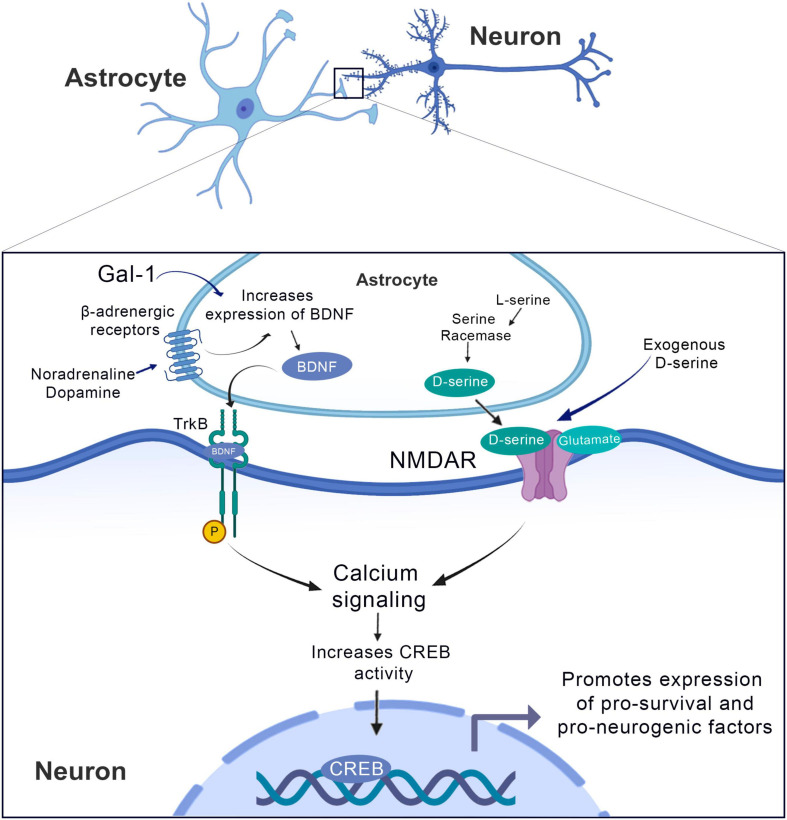
The brain-derived neurotrophic factor (BDNF) supports neurogenesis in normally non-neurogenic brain regions. Gal-1 treatment induces the production of BDNF from cortical astrocytes from this increase in BDNF, an activation of the TrkB receptor occurs, which in turn increases calcium signaling and CREB activity, promoting the expression of pro-survival and pro-neurogenesis factors. Similarly, we observed the effect of noradrenaline and dopamine by increasing the BDNF and following the cascade downstream. In turn, the effect of D-serine is on NDMA receptors, increasing the intracellular calcium signaling favoring the increase of CREB activity.

Gomez-Casati et al. (2010) also identified the synaptogenic potential of BDNF produced by astrocytes. Genetically modified mice, in which the erbB signaling was eliminated in the support cells, failed to form synapses between the hair cells and the sensory neurons of the vestibular organ, it was also shown that this support showed reduction of BDNF and when these cells re-expressed BDNF synapses were recovered. These results show *in vivo* the relevance of erbB and BDNF receptors for vestibular synaptogenesis (Gomez-Casati et al., 2010). In addition, BDNF causes a rapid increase in intracellular calcium through the Trk-PI3K (phosphoinositide 3 kinase) pathway that leads to the formation of a dendritic column (Amaral and Pozzo-Miller, 2007; Figure 5).

The importance of this neurotrophin in neurorepair was highlighted by Béjot et al. (2011). They demonstrated that ischemic stroke models induce an increase in BDNF protein levels 8 days after infarction (Béjot et al., 2011). In accordance, BDNF was also increased in reactive astrocytes 1 day after middle cerebral artery occlusion transitory (tMCAO) in a different report (Zamanian et al., 2012).

Besides, de Pins et al. (2019) have shown that BDNF released by astrocyte increases spine density and dendritic growth in transgenic mice model of Alzheimer’s disease (AD), which is accompanied by cognitive improvements on these animals. In another study by Vignoli et al. (2016), the importance of this neurotrophin was also highlighted. They showed that the interruption of astroglial processing and BDNF secretion leads to deficits and the interruption of memory processes, demonstrated by conducting object recognition behavior tests (Vignoli et al., 2016).

## Astrocyte-Based Strategies to Promote Neurorepair

### Astrocyte-Driven Neurogenesis

As previously discussed, one of the most important signaling pathways correlated with adult neurogenesis is the Wnt/β-Catenin pathway. Hence, one therapeutic approach to enhance adult neurogenesis correlated with astrocytes function is the use of drugs that have Wnt/β-Catenin pathway as a target. For instance, Shruster et al. (2012) have demonstrated a positive effect of Wnt/β-Catenin-related astrocytic-driven neurogenesis using gene therapy. Using injections of lentivirus expressing Wnt3a-HA (LV-Wnt3a-HA), the authors have demonstrated that Wnt/β-Catenin activation can promote enhanced functional recovery after ischemic injury and increase the number of immature neurons in the SVZ and striatum. Moreover, the authors have shown that the neurogenesis enhancement was accompanied by reduced propagation of the neuronal injury.

Furthermore, another treatment that has demonstrated a positive effect on Wnt/β-Catenin-related astrocytic-driven neurogenesis is simvastatin administration. Robin et al. (2014) have demonstrated that oral simvastatin treatment enhances Wnt signaling in the adult hippocampus, increasing the number of newborn neurons in the dentate gyrus through enhancement of intermediate precursor cells (IPCs) in the subgranular zone. These results are in accordance with previous evidence that the statins pharmacological class can increase neurogenesis in the dentate gyrus and reduce delayed neuronal death in CA3 hippocampal region (Lu et al., 2007). Moreover, whereas Wu and collaborators shows that simvastatin-related neurogenesis is mediated by the upregulation of pro-survival environmental changes created with the upregulation of trophic factors such as BDNF and vascular endothelial growth factor (VEGF) (Wu et al., 2008). Robin et al. (2014) demonstrate that simvastatin actions are correlated with Wnt signaling enhancement by depleting isoprenoids, rather than through a cholesterol-dependent mechanism (Figure 5).

There is also evidence on the role of this pathway from a nigrostriatal neurodegeneration model, used to mimic the pathology of PD. In this model, MPTP (1-methyl-4-phenyl-1,2,3,6-tetrahydropyridine) is administered to mice. It is converted by astrocytes to the final toxic agent, MPP+ (1-methyl-4-phynylpyridinium), an inhibitor of the mitochondrial respiratory chain (Efremova et al., 2015; Schildknecht et al., 2015). The oral administration of the NO-donating non-steroidal anti-inflammatory drug flurbiprofen (NO-flurbiprofen) was shown to be neuroprotective in this model. L’Episcopo showed that the damage involves attenuated Wnt signaling via upregulation of GSK-β and β-catenin degradation. Under these conditions, the neurogenic effect of Wnt signaling was reduced. Inhibition/silencing of GSK-3β, Wnt1 exposure, or astrocyte coculture restored neurogenesis in MPTP-treated mice (Figure 4). In fact, treatment with NO-flurbiprofen can activate Wnt/β-Catenin signaling, resulting in GSK-3β downregulation and consequently reduction of the inflammatory SVZ microenvironment in PD. Thus, this promotes neuroprotective effects on neural progenitor cells against mitochondrial dysfunction and cell death, enhancing proliferation and neurogenesis in the SVZ.

As discussed previously, astrocytes manage the microenvironment of synapses by secreting several growth factors such as BDNF, Fibroblast growth factor-2 (FGF-2) and VEGF (Wang et al., 2011; Egervari et al., 2016). Many studies have developed strategies to promote the upregulation of BDNF synthesis and secretion to promote synaptic plasticity and neurogenesis. In fact, Benraiss et al. (2001) showed that adenoviral injection with BDNF constructs in the SVZ is capable of increasing the BDNF in several brain areas outside the hippocampus, such as the olfactory bulb and the striatum. Moreover, intracerebroventricular injection of BDNF leads to an increase in newborn neurons in several areas adjacent to the ventricles, such as striatum, septum and thalamus (Pencea et al., 2001).

Besides the injection of exogenous BDNF, there are relevant data that Galectin-1 (Gal-1), a soluble carbohydrate-binding protein that is widely expressed in both neurons and glia, can enhance astrocytic BDNF production and improve functional outcomes in rats following brain ischemia. In fact, Qu et al. (2010) have demonstrated that exogenous Gal-1 treatment induces the production of BDNF from cortical astrocytes *in vitro* and *in vivo*, which may contribute to brain ischemia through a neurogenic process (Figure 5). Using a rat model of focal ischemia induced by photochemical injury, the authors have shown that 7-days continuous infusion of Gal-1 into subarachnoid space promoted long-term improvement in neurological outcomes along with cell death reduction (Qu et al., 2010). This protective role of Gal-1 was also proved important in another rodent model of focal ischemia, where treatment with this compound was responsible to promote functional recovery and facilitation of neurogenesis (Ishibashi et al., 2007).

Moreover, another treatment that increases astrocytic BDNF synthesis is stimulation of adrenergic receptors. These play several important roles on astrocytes (Christiansen et al., 2011) and treatment of rodent astrocyte cultures with β-adrenergic agonists markedly increases BDNF expression (Zafra et al., 1992; Schwartz and Nishiyama, 1994; Figure 4). Furthermore, Jurič et al. (2006) have demonstrated that noradrenaline and 5-hydroxytryptamine (5-HT) are able to induce an increase in astrocytic BDNF levels in cortical astrocytes (Jurič et al., 2006). Regarding the mechanisms underlying the increase in BDNF levels after noradrenergic stimulation, the authors have demonstrated that the stimulatory effect was abolished by either β1 or β2 selective antagonists, thus confirming the important activity of these receptors on this effect (Jurič et al., 2008). The authors speculate that the stimulation of β-adrenergic receptors leads to the activation of cAMP/PKA cascades, thus leading to an increased phosphorylation of cAMP response element binding protein (CREB), a transcription factor involved in the production of BDNF. Additionally, α-adrenergic receptors stimulation can also promote an increase in astrocytic BDNF levels, presumably by the activation of the PLC-mediated pathway with further activation of calcium-dependent kinases such as Ca^2+^/calmodulin-dependent protein kinase (CaMK) that targets CREB to phosphorylation (Figure 5).

Another important growth factor for promoting astrocyte-driven neurogenesis is the CNTF. CTNF is produced and secreted by astrocytes in neurogenic niches and can promote adult neurogenesis, increasing proliferation and maintenance of newborn neurons (Carroll et al., 1993; Emsley and Hagg, 2003). In fact, there is evidence that exogenous application of CNTF in mice can promote proliferation of neural precursor cells in both neurogenic areas (SVZ) and dentate gyrus (Emsley and Hagg, 2003). The authors hypothesize that this observed effect is correlated with the activation of CNTF receptor, widely distributed on these neurogenic niches. Interestingly, recent studies have shown novel approaches to increase CNTF levels by pharmacological inhibition of focal adhesion kinase (FAK) and Janus kinase (JAK) downstream signaling cascade (Jia et al., 2018). Indeed, the authors have demonstrated that systemic FAK and intrastriatal JNK inhibition enhances SVZ neurogenesis entirely through CNTF, whereas astrocyte-specific deletion of FAK can lead to an increase in CNTF and SVZ neurogenesis as well. Thus, this FAK-JAK-CNTF pathway seems to be a useful therapeutic target to promote astrocytic-driven neurogenesis.

Besides the secretion of growth factors that are crucial to the management of the synaptic microenvironment, another class of molecules secreted by the astrocytes that are also important to this control is the gliotransmitters. As described earlier, an important gliotransmitter secreted by astrocytes surrounding excitatory synapses is D-serine, a neural modulator that acts as an co-agonist of the NMDA receptor in glutamatergic synapses (Schell et al., 1995). Interestingly, since the modulation of glutamatergic synapses can lead to an increase in neuronal activity in neurogenic niches such as the dentate gyrus, D-serine can also exhibit this ability to promote adult hippocampal neurogenesis. In fact, as demonstrated by Sultan et al. (2013), 8 days intraperitoneal administration of D-serine increased cell proliferation and adult hippocampal neurogenesis. Moreover, the authors also have demonstrated that D-serine *in vitro* treatment increased cell number and survival in adult hippocampal neural progenitors suggesting a direct effect of D-serine on adult neural stem cells. The authors hypothesize that this effect in newborn neurons is correlated with an increase in NMDA receptors activity, given that much evidence points to glutamate synaptic activity as responsible for this effect (Platel et al., 2010; Kelsch et al., 2012). Furthermore, besides its effects on neurogenesis, exogenous D-serine has important effects on dendritic spine maturation being relevant for the functional integration of newborn neurons. Sultan et al. (2015), using two distinct conditional transgenic mice to manipulate exocytosis from astrocytes during the maturation stage of new neurons, found that this blockage leads to a markedly impairment on dendritic maturation of newborn neurons, which was partially alleviated by the administration of exogenous D-serine. Thus, modulation of D-serine levels on astrocytes can be a useful target to promote neurogenesis.

### Astrocyte-Driven Synaptogenesis

Astrocytes are key components of the tripartite synapse, being crucial to the energetic balance as well as to synaptic formation and maturation. Astrocytes secrete several factors that contribute to the synaptic plasticity and synaptogenesis, and these can be used in therapeutic approaches to enhance astrocyte-driven synaptogenesis in the brain (Dityatev and Rusakov, 2011). As discussed above, one important factor that is released from astrocytes and can enhance presynaptic formation and transmitter release, contributing to dendrite development is the cholesterol-bind Apolipoprotein (Mauch et al., 2001; Goritz et al., 2005). In fact, Goritz et al. (2005) have demonstrated that astrocyte-secreted cholesterol is crucial for dendritic differentiation, continuous synaptogenesis and functional stability of evoked transmitter release.

**FIGURE 6:**
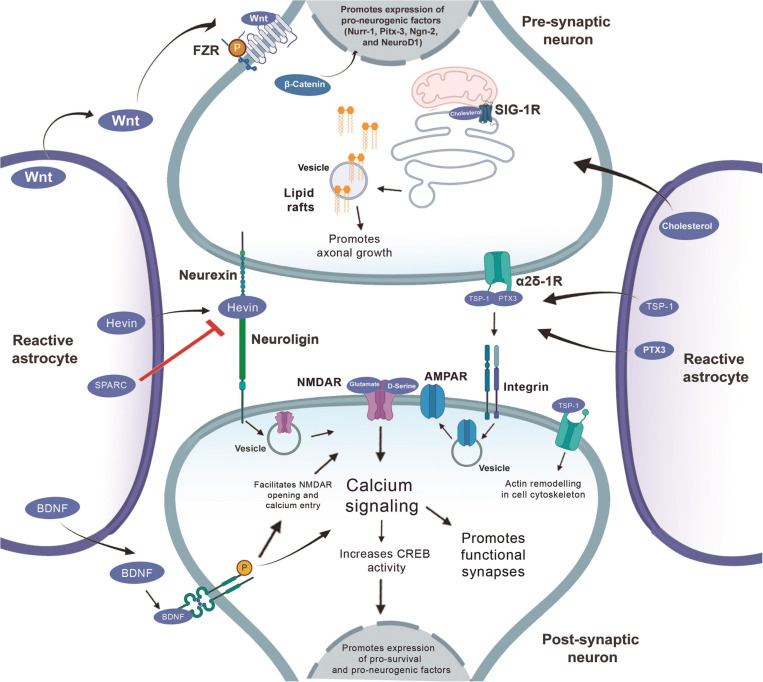
Role of reactive astrocytes in neurorepair in the context of injury and the neurogenic and synaptogenic factors involved in this process. Wnt secreted by the reactive astrocyte interacts with the Frizzled receptors (FZR) and activates the *b*-catenin that will act inside the nucleus promoting the expression of pro-neurogenic genes (Nurr-1, Pitx-3, Ngn-2, and NeuroD1). BDNF activity at TRkB receptors increases calcium signaling, both by increasing CREB activity which, by acting on the nucleus, also promotes the expression of pro-survival and pro-neurogenesis factors, in addition to promoting the formation of functional synapses. D-serine acts similarly via NMDA receptor and CREB activation to promote such factors. TSP1 and PTX3 bind to the α2δ1 receptor, this binding at the presynaptic site increases the number of synapses, but they are silent, so the PTX3 molecule released together with TSP promotes the functional maturation of these synapses through the recruitment of AMPA receptors. The binding of TSP to postsynaptic receptors, activates the Rac1 protein and stimulates the remodeling of actin to promote synaptogenesis. Hevin makes the connection between neurexin and neuroligin and, thus, increases the recruitment of the PSD95 and NMDAR subunits. Hevin’s effect is antagonized by SPARC. The cholesterol released by the astrocyte binds to the sigma-1 receptor and promotes axonal growth.

Moreover, one important target for astrocyte-secreted cholesterol is the Sigma-1 receptor, which is bound to lipid rafts in complex with glucose-related protein 78/binding immunoglobulin protein (GRP78/BiP), an endoplasmic reticulum (ER) chaperone of the mitochondria-associated ER membrane (MAM) domain (Hayashi and Su, 2007). Several Sigma-1R agonists have demonstrated neuroprotective and synaptogenic effects. For instance, stimulating Sigma-1Rs in PC12 cells with ifenprodil or donepezil enhanced neurite outgrowth, whereas the inhibition or knockdown of Sig-1R abolished this synaptogenic effect (Ishima et al., 2008; Ishima and Hashimoto, 2012). Furthermore, another important class of Sig-1R agonists are neurosteroids, such as dehydroepiandrosterone (DHEA), the most abundant endogenous neurosteroid in the CNS (Su et al., 1988). In fact, Hajszan et al. (2004) have demonstrated that subcutaneous injections of DHEA (1 mg/d for 2 days) increased CA1 spine synapse density in ovariectomized rats, which can partially be attributed to the agonistic effect of this compound in Sig-1 receptors, although other unknown mechanisms have not yet been elucidated (Figure 6).

Moreover, there is evidence suggesting that indirect enhancement of cholesterol trafficking to the brain can also promote further upregulation of Sigma1-R activation leading to a marked increase in synaptogenesis and dendritic spine outgrowth. In fact, Cui and colleges have demonstrated that treatment with GW3965, a synthetic liver X receptor agonist, a member of the nuclear receptor family of transcription factors, elevates high-density lipoprotein cholesterol (HDL-c) and promotes increased synaptic protein and axonal density. This effect is partially explained by Sig-1R agonism of cholesterol, although another mechanism could be related with the activation of liver X receptor (LXR), given that LXR activation increase cerebral blood flow and influx of energy substrates into the brain and increase the clearance of toxic products, maintaining a pro-synaptogenic microenvironment (Sandoval-Hernández et al., 2016).

Another important astrocyte-secreted molecule that modulates synaptogenesis is the TGF-β. As discussed above, TGF-β signaling cascade can regulate the formation of excitatory and inhibitory synapses in the brain. Notably, Diniz et al. (2012) have demonstrated that TGF-β signaling pathway is involved in astrocytes synaptogenic modulation through a D-serine dependent cascade. Using treatment of cortical neurons and astrocytes with exogenous TGF-β, the authors have demonstrated an increase on extracellular levels of D-serine, which can modulate NMDA receptors activity and further promote markedly modulation of synaptogenesis and synaptic plasticity. Interestingly, the authors have demonstrated that pharmacological inhibition assays for serine racemase and D-serine abolishes this TGF-β/D-serine synaptogenic effect. Thus, TGF-β signaling pathway can be a useful therapeutic approach to promote synaptogenesis (Figure 6).

Moreover, besides the secretion of TGF-β, another important astrocyte-secreted molecule that is crucial to pro-synaptogenic effect is BDNF. BDNF plays a major role in several cellular processes such as growth, maturation and maintenance of neuronal cells in the brain (Miranda et al., 2019). Furthermore, BDNF can be secreted by astrocytes in an activity-dependent manner, promoting pro-synaptogenic changes in the synaptic cleft microenvironment, mostly from excitatory synapses such as the glutamatergic ones (Gomez-Casati et al., 2010). Hence, therapeutic strategies focused on increasing BDNF levels in the brain can be useful in promoting synaptogenesis. Indeed, several studies have demonstrated a positive effect of BDNF direct injection, gene transduction or delivery via non-viral carriers in different animal models of PD. The treatment could promote not just pro-synaptogenic signaling but also neuroprotective signals that prevented the loss of dopaminergic neurons in the substantia nigra, a crucial part to the development of PD pathogenesis (Tsukahara et al., 1995; Hung and Lee, 1996; Klein et al., 1999; Sun et al., 2005; Hernandez-Chan et al., 2015).

Besides that, there is evidence that suggests a marked increase in BDNF levels in the brain after treatment with antidepressants that selectively inhibits the reuptake of serotonin (called “SSRI”). In fact, Einoch et al. (2017) have demonstrated that treatment with the SSRI fluvoxamine increased the activity of the BDNF-CREB pathway in rat prefrontal cortex, hippocampus and *in vitro* cortical neuronal cultures, whereas the inhibition of PI3K abolished this positive effect. Hence, novel strategies focusing on increasing BDNF levels in the brain may be an interesting approach to promote synaptogenesis (Figure 6).

### Astrocyte-Related Therapeutic Strategies to Induce Neurorepair

In the last 10 years, cell lineage reprogramming has emerged as a novel opportunity to drive cellular regeneration. Initial studies made use of pluripotent stem cells, that were then differentiated to neurons or astrocytes (Takahashi and Yamanaka, 2006; Kim et al., 2011; Han et al., 2012; Ring et al., 2012; Chandrasekaran et al., 2016; Marx et al., 2020). Then, it was found that cell types can be directly transdifferentiated to the neural lineage and that astrocytes in brain may convert into neurons (Vierbuchen et al., 2010; Robel et al., 2011; Ladewig et al., 2013; Kim et al., 2014; Flitsch and Brüstle, 2019; Gatto et al., 2021). Mechanisms found in cell culture were also transferred to transgenic mouse models: direct expression of reprogramming genes in striatal astrocytes converted them into fully functional neurons (Torper et al., 2013). This creates an interesting approach to promote astrocyte-related neurorepair, given the fact that in the injury context, inducing astrocyte-to-neuron trans-differentiation can lead to neural tissue repair.

Furthermore, several molecules have been used to promote astrocyte-to-neuron conversion. For instance, Heinrich and collaborators showed that increasing of Neurog2 and Dlx2 in cortical astrocytes, both transcription factors that are related to neuronal cell-fate of neural progenitors, can produce glutamatergic and GABAergic neurons (Heinrich et al., 2010). Additionally, Guo et al. (2014) have demonstrated that reactive astrocytes of an AD mouse model can be directly reprogrammed to functional neurons *in vivo* using retroviral NeuroD1, another transcription factor related to neuronal lineage. Interestingly, the authors showed that reactive astrocytes were mainly reprogrammed into glutamatergic neurons after NeuroD1 translation.

Rivetti di Val Cervo et al. (2017) have demonstrated the induction of functional dopamine neurons from astrocytes. Using three transcription factors, NEUROD1, ASCL1 and LMX1A, and the microRNA miR218, the authors have demonstrated direct reprogramming of both human astrocytes *in vitro* and mouse astrocytes *in vivo* into dopaminergic neurons. Interestingly, the reprogramming efficiency *in vitro* was improved by administration of small molecules that activate TGF-β and Wnt signaling pathways. Hence, this indicates that modulating both astrocyte-to-neuron conversion and activation of pro-neurogenic pathways is an interesting approach to induce neurorepair.

Another interesting strategy to promote neurorepair that is also related to cell reprogramming is the conversion of astrocytes into neuroblasts to further induce new neurons formation, a process that is called “dedifferentiation” (Kleiderman et al., 2016a). Several *in vitro* studies have demonstrated that many astrocytes in response to injury can re-enter in the cell cycle and differentiate itself in neuroblast, which then is destined to form new neurons (Buffo et al., 2008; Yang et al., 2009; Robel et al., 2011). Promoting adult astrocytes dedifferentiation involves several different pathways, epigenetic silencing of mature astrocyte genes and removement of silencing marks from progenitor genes to further promote a pro-neuroblast cell environment (Robel et al., 2011). For example, increased acetylation of H3K9K14 near NeuroG1 and NeuroG2 genes is required during astrocyte dedifferentiation in mouse primary neuronal cultures (Hirabayashi et al., 2009).

Interestingly, several pieces of evidence have shown that altering the production of many transcription factors and downstream targets can change astrocyte fate and induce dedifferentiation. For instance, Nanog Homeobox, POU Class 5 Homeobox 1 (OCT4), Forkhead Box G1 (FOXG1), SRY-Box 2 (SOX2), and Cell Cycle Exit and Neuronal Differentiation 1 (CEND1) are transcription factors that during situations with increased activity can promote dedifferentiation of adult astrocytes into NSC (Corti et al., 2012; Niu et al., 2013; Aravantinou-Fatorou et al., 2015). Hence, cell reprogramming either by direct astrocyte-to-neuron conversion or astrocyte dedifferentiation into adult neural stem cells is a promising therapeutic approach to promote neurorepair in the context of injury, being crucial on the development of new research to further investigate and elucidated the mechanisms by which these therapies works and its usability in regeneration medicine.

## Conclusion

Astrocytes play a central role in the neurorepair process. They are not only responsible for cleaning the system after injuries and controlling the inflammatory process, but they are also responsible for orchestrating the process of neurogenesis and synaptogenesis. Astrocytes are widely spread throughout the cerebral tissue and can be regenerated after brain damage. Their privileged location in the brain puts them in contact with neurons and endothelial cells, which allows them to control the neuronal differentiation, the synaptic sprouting, and its integration in the networks. Advances in understanding the mechanisms associated with astrocyte activation and its impact on the neurorepair process are fundamental for the development of new therapeutic approaches for astrocyte-induced neurogenesis.

## Author Contributions

RC, GC, BM, LM, OO-L, RMG, FK, FS, AB, and MP wrote the manuscript. RG, ML, and MP revised and approved the manuscript. All the authors contributed to the article and approved the submitted version.

## Conflict of Interest

The authors declare that the research was conducted in the absence of any commercial or financial relationships that could be construed as a potential conflict of interest.
